# From rhetoric to reality: an empirical audit of athlete-centred governance in the Toronto 2015 Pan/Parapan American games

**DOI:** 10.3389/fspor.2026.1864922

**Published:** 2026-07-15

**Authors:** Nicole Wendy Forrester, Eric MacIntosh

**Affiliations:** 1 RTA School of Media, The Creative School, Toronto Metropolitan University, Toronto, ON, Canada; 2 School of Human Kinetics, Faculty of Health Sciences, University of Ottawa, Ottawa, ON, Canada

**Keywords:** athlete experience, athlete satisfaction, athlete-centred, experiential parity, mega-sport event, prosumer, sport governance, stakeholder salience

## Abstract

In contemporary mega-sport events (MSEs), athlete-centred governance has become a dominant rhetorical commitment, yet it is often operationalized through service delivery rather than formal governance structures. Within the stakeholder identification and salience framework, athletes are typically positioned as dependent stakeholders, characterized by high legitimacy and urgency but limited formal power. Despite functioning as prosumers, athletes rarely possess meaningful decision-making authority. This study examines athlete-centred governance by assessing whether the formal integration of an Athlete Advisory Council (AAC) within an MSE is associated with athlete satisfaction. Using a cross-sectional, field-based design, this study examines the Toronto 2015 Pan/Parapan American Games. A total of 829 athletes representing 42 sports and 29 countries completed the athlete experience questionnaire (AEQ). Strong internal consistency was observed (Cronbach's *α* = .84–.92), and AAC-identified factors were significantly associated with satisfaction (*p* < .001). Multiple regression analysis indicated that governance factors identified by the AAC explained 83.8% of the variance in satisfaction (*p* < .01), with social aspects (*β* = .575, *p* < .001) and communication (*β* = .324, *p* < .001) being the strongest positive predictors. Additionally, findings demonstrate experiential parity across diverse athlete cohorts. Results suggest that formalizing and operationalizing athlete voice within MSE structures is associated with athlete-centredness.

## 1 Introduction

There has been a rising trend for mega-sports events (MSEs) to promote an athlete-centred approach (1–4). However, its application remains inconsistent. Despite widespread adoption, there is limited consensus on how athlete-centredness should be defined or operationalized. Recently, the International Olympic Committee's (IOC) Olympic Agenda 2020 + 5 identifies athlete-centredness as inclusive of athlete well-being and safety, while also advocating for the representation of athletes within sport (1, 5). Similarly, the 2026 FIFA World Cup frames its approach as a commitment to player welfare and enhancing the player experience at tournaments (6). Comparatively, through athlete representation, prioritizing the athlete experience at a Games, and promoting equality and inclusion, Commonwealth Sport positions itself as an athlete-centred organization (7). While interpretation of athlete-centredness may vary across MSEs, athletes are often treated as passive objects of service delivery rather than active subjects in governance (2, 8–10). Athlete-centredness is often defined as a service delivery philosophy focused on athlete experience or as a governance principle that emphasizes athlete representation in decision-making. This ambiguity limits its practical application, as outcomes are typically assessed through service quality rather than structural inclusion in governance processes. This paper addresses this gap by reconceptualizing athlete-centredness in MSEs as a matter of formal sports governance, prioritizing the systemic inclusion of athlete voice in decisions directly affecting them.

Of all the key stakeholders in an MSE, athletes are the only group that simultaneously functions as both producers of the event and its primary consumer. Accordingly, they can be understood as prosumers (11, 12). However, despite growing recognition of their centrality to both the production and legitimacy of MSEs, athletes have historically lacked decision-making authority and remain largely confined to consultative roles (10, 13–16). This places them in a contradictory position. While they are essential to producing an event, their role as consumers is institutionally prioritized (17). Reconceptualizing athletes as partners in the decision-making process requires moving beyond a service delivery perspective toward understanding athlete-centredness as a governance process (8, 18). Yet in practice, athlete-centredness is often treated as a normative ideal rather than examined as an embedded aspect of governance.

By applying stakeholder theory, this study examines how MSEs prioritize stakeholders in decision-making. Specifically, Freeman (19, 20) posits that the long-term success of an organization depends on its ability to balance stakeholder needs. While athletes may be identified as a key stakeholder, their role in an MSE's decision-making process is not well understood. This study therefore adopts the framework of stakeholder identification and salience (21). Within this framework, stakeholders are prioritized according to the interrelated attributes of power, legitimacy, and urgency, which together determine their influence within organizational decision-making processes. Athletes are typically classified as a dependent stakeholder possessing urgency and legitimacy, but lacking power (22). Applied to MSEs, a structural imbalance becomes apparent (1, 18, 23, 24). Because MSEs cannot occur without athletes (legitimacy), their performance and well-being demand timely attention (urgency). Nonetheless, they generally have limited formal power within organizing committees. As a result, athletes must rely on others to act on their behalf (25). Comparatively, administrators, sport organizations, and external experts occupy dominant or definitive positions, wielding decision-making power in an MSE landscape, even though they carry less inherent legitimacy. This imbalance helps explain why athletes often remain central only in rhetorical terms (26). Despite these theoretical insights, there is limited empirical evidence examining whether governance structures that incorporate the athlete voice are associated with measurable athlete outcomes in MSEs. The present study examines athlete experience as an outcome associated with governance conditions. The analysis focuses on athlete satisfaction as a system-level outcome of the governance environment rather than the specific decision-making processes through which athlete-centred governance was constructed and enacted. These governance processes are examined separately through process-oriented research (27).

To address this gap, the Toronto 2015 Pan/Parapan American Games (TO2015) provide a unique empirical case. As early as the bid process, organizers identified a goal of having an athlete-centred Games. In support of this, an Athlete Advisory Council (AAC) was formally established by the organizing committee. Athletes representing various sports, including both para and able-bodied athletes, were intentionally selected (28). This governance structure positioned athletes as both stakeholders and contributors (12). As a result, TO2015 represents a particularly informative case through which to examine athlete-centred governance in practice. Through this organizational structure, the AAC had the unique opportunity to share their perspectives on decisions directly affecting participating athletes (27). This operationalization of the council illustrates the application of stakeholder theory. However, while athletes held legitimacy and urgency as primary contributors to the Games experience, they lacked formal power within TO2015, as they did not have voting authority on these decisions.

To implement an athlete-centred approach within TO2015, the AAC was integrated into the organizing committee's structure, as a formal means to embed athlete voice in the planning and organizing of the event (27, 29). National team athletes across nine different events, with varying professional backgrounds (e.g., medical, communication, business) representing both able-bodied and para-athletes were appointed by the organizing committee. Through this diverse skillset and background, athletes were able to provide expert knowledge to decisions affecting the environment of athletes, from competition scheduling and transportation logistics to housing and medical services. While prior research has explored how athlete-centred governance structures are designed (27), less is known about whether these structures are associated with measurable outcomes for athletes. By incorporating council members across multiple levels of planning and decision-making, TO2015 created opportunities for athletes to shift from dependent stakeholders to active contributors whose perspectives were valued. While the structural aspects of the athlete-centred governance model are examined in a separate study (27), this study investigates whether this governance approach was associated with positive athlete experiences and satisfaction. Nine functional areas shaping the athlete experience were identified by the AAC in their formal role. These included atmosphere, accommodation, transportation, security, ceremonies, technology, food, communications, and volunteers. Within each domain, AAC representatives participated in working groups, and provided feedback on operational plans (28).

The AAC's involvement in the TO2015 illustrates mechanisms for balancing stakeholder power within an MSE. By formalizing meeting schedules, decision-making protocols, and reporting lines, the athlete voice was active and actionable (e.g., white paper). Under the stakeholder identification and salience framework, TO2015 enhanced athletes’ stakeholder salience by institutionalizing avenues of influence through the AAC, recognizing their inherent legitimacy, and responding to urgent concerns through feedback loops. These mechanisms were designed to address the traditional marginalization of athletes in MSE governance (14). Building on this gap, the present study conceptualizes athlete-centred governance as a multilevel process linking organizational structure to athlete outcomes. The framework proposes that governance structures (e.g., formal integration of an AAC), decision-making processes, athlete-facing services, and athlete experiences are interconnected components of an athlete-centred environment. Accordingly, this study examines whether the formal integration of an AAC within TO2015 was associated with measurable athlete satisfaction across key service areas. It moves beyond rhetorical and normative ideals of athlete-centredness to provide an empirical assessment of how athlete-centred governance is associated with athlete experience within an MSE. This provides a rare empirical audit of athlete-centred governance within an MSE context.

## 2 Method

### 2.1 Participants

Upon institutional review board approval and permission of the TO2015 organizing committee, athletes competing in TO2015 were purposively sampled to participate in the study. A total of 829 athletes participated in the study, including 377 female (45.5%), 434 male (52.4%), and 18 who did not report gender. Twenty-nine additional questionnaires were excluded due to incomplete responses. Participants included 538 able-bodied athletes (65%) and 273 para-athletes (33%), with a small proportion not reporting classification. Athletes represented 29 countries and 42 sports. Participants included 485 athletes (58.5%) from team sports, 237 (28.6%) from individual sports, and 90 (10.9%) who competed in both. A total of 539 participants (65%) reported prior multi-sport event experience, while 269 (32.4%) were competing in their first major Games. Surveys were completed in English (*n* = 405, 48.9%), Spanish (*n* = 413, 49.8%), and French (*n* = 11, 1.3%), reflecting the trilingual policy of the TO2015 organizing committee.

### 2.2 Instrumentation

The athlete experience questionnaire (AEQ), a previously validated multidimensional instrument used in multi-sport event contexts (12), was adapted to reflect the service delivery environment of TO2015. Minor contextual wording modifications were made while maintaining the conceptual integrity of the original scale. The TO2015 AAC reviewed the questionnaire as subject matter experts and confirmed that the items reflected the controllable service factors prioritized by council members (12). As the AEQ was previously found to have acceptable factorial validity in a comparable multi-sport event context, and only minimal modifications were needed to adapt the measure for TO2015, no further factor analysis was performed on the current sample. Structural coherence was assessed using internal consistency indices, inter-factor correlations, subject matter expert review, and prior psychometric support. Although this approach does not provide direct confirmation of the factor structure within the present sample, it is consistent with established field-based practices for time-sensitive, large-scale event research (30–32).

The survey consisted of 32 items across six domains: (1) basic needs (e.g., bed quality and nutrition); (2) services (e.g., arrival, accreditation, transportation); (3) communication (e.g., wireless, administrative support, Games handbook); (4) social aspects (e.g., opening ceremony and Village atmosphere); (5) competition needs (e.g., competition management and access to appropriate training equipment); and (6) satisfaction. Items were measured on a 6-point Likert-type scale (1 = very poor, 2 = poor, 3 = satisfactory, 4 = good, 5 = excellent; and 6 = not applicable). Responses were treated as approximately continuous to permit parametric analyses, consistent with prior research using Likert-type scales in sport management contexts. “Not applicable” responses were treated as missing and excluded using pairwise deletion, resulting in varying sample sizes across items (33). Open-ended questions were included to capture additional athlete perspectives.

### 2.3 Procedure and data collection

This study employed a field-based, cross-sectional design. Athletes were recruited within the Athletes' Village dining hall and social areas. Surveys were administered in both hard-copy and digital formats to accommodate athlete schedules. The recruitment documents and questionnaires were available in English, Spanish, and French. Participants received a written explanation of the study, including their rights and ability to withdraw at any time, and provided informed consent prior to participation. Data were collected from the first day of competition until four weeks post-Games to allow for both immediate feedback and retrospective reflection. Participants were entered into a draw for a $30 gift certificate.

### 2.4 Data analyses

Quantitative data were analyzed using SPSS to compute descriptive statistics (i.e., means, standard deviations, and frequencies) and the psychometric properties of the AEQ factors. Assumptions of normality, homogeneity, and multicollinearity were satisfied. Variance inflation factor (VIF) scores were within acceptable limits (33). Multiple regression and one-way analyses of variance (ANOVA) were conducted to examine factors associated with athlete satisfaction and group differences. Pairwise deletion was used to retain available data across items (34).

Open-ended responses were analyzed to provide contextual insight into athlete perceptions. Responses were analyzed using a descriptive thematic analysis by two authors, who independently reviewed all responses to identify recurring themes. Themes were identified through grouping similar responses. Representative quotations were selected to illustrate or explain quantitative data. Qualitative findings were used to complement quantitative results. Given the brevity and optional nature of the responses, this approach was sufficient for descriptive purposes.

### 2.5 Trustworthiness

Trustworthiness was established through peer-debriefing, methodological triangulation, and the maintenance of an audit trail (35). Peer debriefing involved iterative discussions between the authors to refine theme identification and interpretation. Methodological triangulation was achieved by cross-referencing the qualitative themes with the quantitative regression and ANOVA results. An audit trail documenting coding decisions and theme development was maintained to ensure transparency and dependability. The lead author's positionality as both an Olympian and vice chair of the AAC offered considerable contextual knowledge of the TO2015 environment. At the same time, this dual role may have introduced a risk of confirmation bias. To reduce the influence of personal assumptions on data interpretation and increase transparency, several safeguards were implemented. This included maintaining reflexive journaling throughout data collection and analysis. Peer debriefing with the second author, who was not involved in the AAC, provided an additional external perspective on coding decisions and interpretation of findings. Furthermore, the quantitative analyses were conducted using pre-specified survey measures and standardized statistical procedures, thereby reducing reliance on subjective interpretation alone. The authors recognize that complete neutrality is neither possible nor necessarily desirable in applied sport research. Accordingly, the purpose of these procedures was not to eliminate researcher influence entirely, but rather to make positionality explicit and enhance transparency, credibility, and analytical rigor throughout the research process.

## 3 Results

Internal consistency and inter-factor correlations were analyzed to assess whether the AEQ captured relevant functional domains. Strong internal consistency was observed across all factors (Table 1) (33). Cronbach's *α* ranged from .84 (services, communication) to .92 (social aspects), while satisfaction had an alpha value of .94, exceeding commonly accepted thresholds (33). The mean scores of all factors were relatively high with the exception of Housekeeping under Services (*M* = 3.71, SD = 1.03).

**Table 1:** Reliability and descriptive statistics for AEQ factors and items.

Factor	Item	*α*	*N*	*M*	SD
Basic needs		**.85**	**815**	**4.40**	**.48**
	Bedroom		826	4.04	.81
	Bathroom		815	4.11	.85
	Food quality		827	4.49	.67
	Food availability		824	4.56	.65
	Water availability		825	4.81	.46
Services		**.84**	**795**	**4.31**	**.50**
	Arrival process		799	4.35	.78
	Accreditation process		821	4.49	.71
	Transportation		814	4.26	.89
	Medical provisions		769	4.47	.66
	Laundry		783	4.22	.87
	Housekeeping		811	3.71	1.03
Competition needs		**.86**	**803**	**4.38**	**.68**
	Competition venue equipment		820	4.51	.73
	Competition management		788	4.39	.76
	Competition venue training		816	4.21	.94
Social aspects		**.92**	**636**	**4.50**	**.51**
	International zone		772	4.26	.83
	Outside of competition		748	4.33	.74
	Venue social environment		791	4.32	.79
	Welcoming ceremony		562	4.36	.76
	Opening ceremony		566	4.38	.76
	Medal ceremony		430	4.47	.67
Communication		**.84**	**754**	**4.28**	**.61**
	Handbook		612	4.10	.79
	Family contact		779	4.20	.83
	Staff contact		791	4.35	.74
	Wi-Fi		816	4.10	.93
Satisfaction (dependent variable)		**.94**	**790**	**4.59**	**.52**
	Games experience		804	4.58	.56
	Family and friends		795	4.63	.56
	Comparative with other events		793	4.55	.63

Includes sample size (*N*), mean scores (*M*), standard deviations (SD), and Cronbach's alpha (*α*) for each factor and survey item. Sample sizes vary due to item-level missing data. Factor score computed using available item responses.

Inter-factor correlations (Table 2) were moderate to large in magnitude (*r* = .43–.71, *p* < .01). Correlations indicated that factors were related but not redundant. No correlations exceeded .90, suggesting multicollinearity was unlikely (33).

**Table 2:** Intercorrelations (*r*) of the AEQ factors.

Factor	1	2	3	4	5	6
Basic needs	–	.65	.71	.54	.55	.58
Services		–	.69	.63	.68	.67
Competition needs			–	.46	.56	.43
Communication				–	.67	.68
Social aspects					–	.58
Satisfaction						–

All correlations significant at *p* < .01.

### 3.1 Regression analysis: predictors of athlete satisfaction

A multiple regression was conducted with the five fundamental domains (basic needs, competition needs, communication, services and social aspects) as predictors of overall athlete satisfaction. The results were statistically significant [*F*_(5, 823)_ = 851.45, *p* < .001] and accounted for 83.8% of the variance in satisfaction (Table 3). VIF values were within acceptable limits (33).

**Table 3:** Multiple regression predicting overall athlete satisfaction.

Factor	*β*	*t*	Sig. (*p*)	VIF
Social aspects	.575	18.84	<.001	2.57
Communication	.324	11.48	<.001	2.05
Basic needs	.301	5.93	<.001	2.52
Services	.125	2.79	.005	2.66
Competition needs	-.210	−10.43	<.001	2.08

*R*^2^ = .838, *F*_(5, 823)_ = 851.45, *p* < .001. Standardized *β* coefficients reported. Variance inflation factors (VIF) < 3.0 indicate that multicollinearity did not bias the regression estimates. Social aspects and communication were the strongest positive predictors of satisfaction, while competition needs had a negative association.

Social aspects (*β* = .575, *p* < .001) and communication (*β* = .324, *p* < .001) had the strongest positive associations with athlete satisfaction. Basic needs (*β* = .301, *p* < .001) and services (*β* = .125, *p* = .005) were also positively associated with overall satisfaction, whereas competition needs were negatively associated (*β* = −.21, *p* < .001).

### 3.2 Group differences: parity of athlete experience

To examine equivalence of experience among athletes, one-way ANOVAs were conducted comparing able-bodied athletes to para-athletes. One-way ANOVAs revealed significant differences in services, *F*_(1, 794)_ = 12.97, *p* < .01, *η*^2^ = .016. The other areas, including basic needs, competition needs, communication, social aspects, and satisfaction, did not differ significantly (all *p* ≥ .05) (Table 4).

**Table 4:** Able-bodied and para-athlete experiences.

Factor	Able-bodied athletes			Para athletes					
	*N*	*M*	SD	*N*	*M*	SD	*F*_(1,df)_	*p*	*η* ^2^
Basic needs	531	4.38	.49	267	4.43	.44			n.s.
Services*	515	4.25	.51	263	4.44	.43	12.97	<.01	.016
Competition needs	520	4.37	.61	267	4.38	.80			n.s.
Communication	488	4.25	.61	249	4.31	.61			n.s.
Social aspects	388	4.53	.51	236	4.46	.51			n.s.
Satisfaction	517	4.60	.51	258	4.51	.58			n.s.

*F*-values reflect one-way analyses of variance comparing able-bodied and para-athletes. Degrees of freedom for the services factor: *F*_(1, 794)_ = 12.97, *p* < .01. *η*^2^ = eta squared effect size. n.s., not statistically significant (*p* ≥ .05). Sample sizes vary due to missing data. The *η*^2^ value indicates a small effect, suggesting a statistically significant but modest difference in service experiences between cohorts, *p < .01 *F*_(1, 794)_.

Similarly, analyses were computed to determine differences between veteran and rookie athletes. One-way ANOVAs did not reveal statistically significant differences in services [*F*_(1,792)_ = 2.97, *p* ≥ .05, *η*^2^ = .004], social aspects [*F*_(1, 633)_ = 3.25, *p* ≥ .05, *η*^2^ = .005], or overall satisfaction [*F*_(1, 789)_ = 3.52, *p* ≥ .05, *η*^2^ = .004]. No statistically significant differences were observed for basic needs, competition needs, or communication (all *p* ≥ .05) (Table 5).

**Table 5:** Rookie and veteran athletes in the multi-sport event environment.

Factor	Veteran athletes			Rookie athletes					
	*N*	*M*	SD	*N*	*M*	SD	*F*_(1,df)_	*p*	*η* ^2^
Basic needs	529	4.40	.48	266	4.41	.46		n.s.	
Services	515	4.30	.52	261	4.36	.43	*F*_(1, 792)_ = 2.97	n.s.	.004
Competition needs	524	4.40	.65	260	4.33	.74		n.s.	
Communication	488	4.27	.60	247	4.29	.62		n.s.	
Social aspects	424	4.47	.53	198	4.58	.44	*F*_(1, 633)_ = 3.25	n.s.	.005
Satisfaction	525	4.56	.54	247	4.66	.48	*F*_(1, 789)_ = 3.52	n.s.	.004

*F*-values reflect one-way analyses of variance comparing rookie and veteran athletes. Degrees of freedom: Services *F*_(1, 792)_ = 2.97, social aspects *F*_(1, 633)_ = 3.25, satisfaction *F*_(1, 789)_ = 3.52. *η*^2^ = eta squared effect size. n.s., not statistically significant (*p* ≥ .05). Sample sizes vary due to missing data. The *η*^2^ values indicate very small effects, suggesting minimal differences between rookie and veteran athletes.

### 3.3 Open-ended responses

Using descriptive thematic analysis, two authors independently reviewed all open-ended responses. Discrepancies were resolved through discussion, and emerging themes were documented. The main themes that emerged included food/cafeteria, accommodations, transportation, performance outcomes, opening ceremony, crowd support, friendly volunteers, meeting new people, entertainment/activities, Wi-Fi, and the Athletes' Village.

## 4 Discussion

Athlete-centredness has become a popularized approach endorsed by various sports organizations and MSEs. While many claim to engage in this practice, its operationalized effect within an MSE is not well understood (9, 16). This study conceptualized and empirically examined athlete-centred governance as a process linking organizational structure to measurable athlete outcomes. The formal integration of the AAC was associated with high athlete satisfaction (*M* = 4.59, SD = .52) and substantial explanatory power within the regression model (*R*^2^ = .838). A key consideration in interpreting these results is the position of the AAC within the broader governance structure. The AAC operated within a broader governance system intended to inform decision-making processes.

Within the stakeholder identification and salience framework (21), athletes are characterized by legitimacy and urgency. In TO2015, power was operationalized through formal and informal integration of the AAC across organizational levels. This provides insight into how athlete-centred governance was enacted within TO2015. Vertical and horizontal integration enabled athlete input to be embedded within planning and operational processes (27). With the commencement of the Games, the transition of AAC members into mayor roles within the Athletes’ Village allowed council members to extend their influence into real-time service delivery (e.g., promoting activities occurring in the village, addressing issues and concerns that arose, and by taking a genuine interest in the athletes and their experience). This operational shift illustrates how athlete-centred governance can be extended beyond planning into implementation. Within the stakeholder identification and salience framework, these administrative and communication functions could be viewed as the means by which athletes gained more influence in the governance process, although they lacked formal voting rights (1, 25, 29). This form of embedded, real-time engagement is consistent with the positive evaluations reported across service domains (Table 1).

Although AAC members did not hold formal voting power, they exercised influence through embedded expertise and operational authority. From this perspective, the TO2015 athlete-centred commitment can be interpreted as a deliberate attempt to prioritize athletes as stakeholders (36). Rather than confirming causality, the findings provide observed evidence that athlete prioritization can be meaningfully incorporated within MSE governance. The findings suggest that, for the 829 participating athletes, the strategic intent of the TO2015 mandate was largely realized.

To better understand the strength and implications of this study, the significance of *R*^2^ = .838 requires careful consideration. Based on conventional benchmarks, this represents a very large effect size. However, such benchmarks are heuristic and should be interpreted within the context of the study design and measurement framework (37). In this case, multicollinearity and shared method variance were likely associated with the high explained variance, given the moderate-to-strong correlations observed among predictors (*r* = .43–.71) (33). Additionally, because overall satisfaction represents a holistic evaluation of the athlete experience encompassed by the five service domains, some degree of conceptual overlap between the predictors and criterion variable is expected. Rather than representing wholly independent constructs, the service domains capture distinct but related dimensions of the broader athlete experience. This relatedness among constructs may help explain the strong associations observed in the regression model.

Within a highly centralized event setting, these variables may not be perceptually distinct for athletes (38), but rather represent an integrated MSE experience. Since all measures were self-reported and collected at the same point in time using a single questionnaire, common method variance may also inflate relationships between constructs. Shared response tendencies, mood states, and overall evaluations of the Games environment may therefore have strengthened observed associations beyond what might emerge from multiple measurement methods or data sources. However, rather than undermining the framework, this relationship may reflect the inherent connectedness of the athlete experience within MSEs where the various dimensions of service are perceptually interconnected. Accordingly, the findings are best interpreted as a convergence of services reflecting athletes' holistic evaluation of the Games experience.

Comparatively, the negative association observed for competition (*β* = −.21, *p* < .001) also warrants careful interpretation. At the bivariate level, competition needs were positively correlated with overall satisfaction (*r* = .43). This indicates that athletes who reported more favourable competition conditions also tended to report greater overall satisfaction. However, when considered alongside the other service domains in the regression model, the coefficient became negative. One possible explanation relates to the expectations of high-performance athletes. For veteran athletes, venues, equipment, scheduling, and technical operations are not optional improvements but fundamental requirements for performance. Having competed in previous MSEs, these athletes may have expected TO2015 to meet world-class standards and provide optimal performance conditions. Consequently, competition-related factors may function more as baseline expectations than as independent sources of satisfaction. Athletes who place greater emphasis on these conditions may therefore apply more stringent evaluative standards when assessing the overall Games experience, resulting in comparatively lower global satisfaction despite favourable competitive environments. This interpretation is consistent with expectation-based perspectives of satisfaction within sport event contexts (38). A possible suppression effect within the regression model may also help explain this finding. As a result, the negative coefficient should not be interpreted as evidence that favourable competition conditions reduce athlete satisfaction, but rather that performance-related aspects of the Games experience may be evaluated differently from other service dimensions.

Athlete-centredness cannot be explained solely through one of the domains, since it depends on the alignment of multiple functional areas. Collectively, these domains appear relevant to both performance-related conditions and athlete experience at the Games. Within this integrated model, the social environment was the strongest predictor of satisfaction. Communication also emerged as a strong predictor, highlighting the importance of both relational and informational aspects of athlete-centred delivery.

A key objective of this athlete-centred governance audit was to examine the extent of experiential parity across athlete groups. While there is a push for major Games hosting both able-bodied and para-athletes to have parity, factors such as venue, media coverage, sponsorship, and crowd attendance can result in an inferior experience for para-athletes (39). Para-athletes reported high evaluations across all functional areas, similar to able-bodied athletes, with only a small statistically significant difference observed in services (*η*^2^ = .016). This seems to suggest that limited practical differences between groups existed. To further illustrate the quantitative findings, open-ended responses from para-athletes reinforced positive aspects of the Games environment with comments such as: “The staff and volunteers have been amazing and very friendly,” “AWESOME STAFF AND VOLUNTEERS!” These comments are consistent with the positive evaluations reported by para-athletes and highlight the perceived value of volunteers and staff within their Games experience. In contrast, negative comments from para-athletes were limited to mainly logistical challenges. For example, some para-athletes described transportation challenges following the opening ceremony and congestion in shared facilities disproportionately. One athlete stated, “Transport leaving the opening ceremony was a bust.” By comparison, able-bodied athletes also expressed some concerns related to transportation as well as housekeeping (items within the service factor). For example, one athlete perceived that, “Maids stole money […] Bus system [was] shaky at times to get to competition venue on time.” Despite this, the results suggest that TO2015 achieved parity in perceived satisfaction between para-athletes and able-bodied athletes, with both reporting a high degree of satisfaction (40). This finding is notable given persistent concerns regarding inequities in para-sport event experiences.

Although differences between rookie and veteran athletes were not statistically significant, descriptive patterns suggested that expectations may shape how athletes evaluate the Games experience (41, 49). One-way ANOVAs revealed no statistically significant differences in services, *F*_(1, 792)_ = 2.97, *p* ≥ .05, *η*^2^ = .004, social aspects, *F*_(1, 633)_ = 3.25, *p* ≥ .05, *η*^2^ = .005, or overall satisfaction, *F*_(1, 789)_ = 3.52, *p* ≥ .05, *η*^2^ = .004. However, rookie athletes reported slightly higher mean scores in social aspects (*M* = 4.58, SD = .44) and satisfaction (*M* = 4.66, SD = .48), whereas veteran athletes reported slightly higher evaluations in competition needs (*M* = 4.40, SD = .65) compared to rookies (*M* = 4.33, SD = .74). These descriptive patterns suggest that different aspects of the Games experience may be emphasized depending on athletes' prior MSE experience. For athletes competing in their first major Games (rookies), the experience may be interpreted through a lens of novelty and discovery. In contrast, veteran athletes likely evaluated their experience relative to previous MSE participation (42, 49). No statistically significant differences were observed in competition needs between groups (all *p* ≥ .05), with veteran athletes reporting slightly lower mean scores for services (*M* = 4.30, SD = .52) and social (*M* = 4.47, SD = .53) domains, compared to rookie athletes (services, *M* = 4.36, SD = .43; social aspects, *M* = 4.58, SD = .44).

This pattern is consistent with expectation-based models of satisfaction. While rookies considered the opening ceremony a “perfect” event, veterans may be more likely to interpret high-quality facilities as a minimum requirement, rather than as a highlight. For rookies, TO2015 appeared to represent a unique and socially engaging experience. By comparison, veteran athletes may place greater value on performance-related and logistical conditions. Taken together, these patterns suggest, cautiously, that athletes' perceived satisfaction may be shaped by the interaction between performance conditions and expectation-based references, rather than service factors alone.

Consistent with these descriptive patterns, rookie athletes provided qualitative feedback that captured the scale of the Games as something new and memorable, stating: “The opening ceremony was perfect,” “[An] opportunity to meet other athletes from every sport,” “Learning, keep it up, taking in all the experience. Learning how it feels like to compete in an event—so how the Games works, personal goals,” “Great precursor experience to the Olympics,” and “Best experience of my life so far!” These statements suggest that rookie athletes may evaluate the Games experience through less firmly established expectations (41), which may contribute to higher evaluations in service and social satisfaction compared to veterans. Although not statistically significant (*p* ≥ .05), competition needs was the only factor for which veteran athletes reported a slightly higher mean score than rookie athletes. In the open-ended answers athletes expressed appreciation for the various competition venues and support of the crowd. For example, athletes stated, “The venue is nice and accessible,” “The track and field stadium is perfect,” “[The] crowd at our venue was a highlight, lots of people.”

Overall, this study offers three contributions to sport policy and governance literature. First, by linking athlete prioritization as a stakeholder to measurable experiential outcomes, the stakeholder identification and salience framework is operationalized within an MSE. Second, the findings suggest that athlete experience in an MSE is systemic and may be better understood as a combination of individual services offered during the Games. Lastly, descriptive patterns suggest that cohort-specific expectations may contribute to differences in how rookie and veteran athletes evaluate the Games experience. Collectively, these contributions demonstrate how athlete-centred governance can be empirically examined through athlete experience outcomes within the MSE environment. An important consideration when interpreting these findings is the unique context of TO2015. Specifically, the Games explicitly committed to an athlete-centred approach and formally integrated an AAC across organizational structures. As such, the findings should not be assumed to generalize automatically to other MSE contexts where athlete representation may be less formalized or less integrated into decision-making processes. Rather, the present study should be viewed as an examination of a governance configuration in which athlete-centred principles were intentionally embedded throughout the event environment.

## 5 Practical implications

From a policy perspective, this research provides empirical support for the feasibility of formally implementing athlete-centred governance within an MSE. At the same time, it serves as a reminder that the effective execution of an athlete-centred approach requires both strategic planning and effective implementation. The alignment between the athlete-centred objectives established by TO2015 and athletes' reported experiences suggests that there was a strong strategic fit. However, the gaps in the service provision indicate that constant monitoring and adaptation are necessary. More broadly, this study illustrates how stakeholder salience can go beyond being a normative framework to becoming a practical governance mechanism within the MSE environment.

Building on these findings, athlete experience appears to be associated with organizational and environmental factors that can be actively managed within the MSE context (43, 44). While sport event research has examined different stakeholders like the customer and sport tourist (45, 46), employees, and volunteers (47, 48) far less attention has focused on the athlete. More broadly, this study contributes to a growing body of literature recognizing the athlete as a central—but historically underexamined—stakeholder in sport event management (42).

## 6 Institutional challenges

While the TO2015 case offers empirical evidence for a structured form of athlete-centred governance within an MSE, several challenges still remain. Athletes' ability to meaningfully influence decision-making was a strength of TO2015. However, organizations may be resistant to incorporating athlete influence into traditionally top-down governance structures. Likewise, there is a persistent risk of tokenism when it comes to athlete representation, whereby athletes exist in name but lack horizontal and vertical integration within an organization. In such instances, athletes' knowledge and experience are not valued, while the illusion of meaningful athlete representation remains. Given the importance of these functional areas to the athlete experience, token forms of athlete representation may be insufficient to support the objective of an athlete-centred event. Going forward, the path towards a gold standard athlete-centred approach will have to involve more than empty rhetoric about inclusion. It should involve the integration of para and able-bodied athletes as prosumers into the co-design process of the MSE environment.

## 7 Future directions

Given athletes' dual role as prosumers, future research should examine how different models of integrating athletes in governance are associated with athlete outcomes across MSE contexts. Specifically, comparative studies could assess whether formal voting power embedded in athlete representation roles or hybrid governance structures result in different implications for athlete experience. Additionally, future research should examine the relationship between athlete experience and performance outcomes. Given the prosumer role of athletes, longitudinal research across the MSE lifecycle could provide greater insight into how governance structures shape both the experiential and performance-related outcomes for the athlete.

## 8 Limitations

When interpreting these findings, several limitations should be noted. Notably, this study relied on athletes' self-reported data, which limits causal interpretation. As such, the results cannot be interpreted as causal relationships, but rather as associations between variables. Given the time-sensitive nature of TO2015 and the fact that this study adapted a previously validated AEQ from a comparable MSE, internal consistency and inter-factor correlations were analyzed as practical indicators of structural coherence. Therefore, a full exploratory or confirmatory factor analysis was not conducted in this study. Although the AEQ had previously demonstrated acceptable factorial validity and only minor contextual adaptations were made, the absence of an exploratory or confirmatory factor analysis limits the ability to fully evaluate whether the original factor structure was replicated within the TO2015 context. Future research should seek to confirm the dimensionality of the adapted instrument across MSE and across linguistic contexts. Likewise, the moderate inter-factor correlations raise the possibility of multicollinearity, whereby the various functional areas may be moderately correlated. These findings may also reflect shared method variance associated with the high explained variance observed in the regression model.

A key limitation of this study is the difficulty of isolating the independent contribution of different governance mechanisms, including the AAC. Since this design does not allow for a causal analysis, the AAC cannot be investigated in isolation. Rather, the research approach is that of a system-level analysis where many different structures and processes coexisted at one point in time. As a result, the findings from this governance configuration should not be interpreted as causally attributable to any particular mechanism.

Although multiple safeguards were implemented to enhance trustworthiness, the lead author's role as Vice-Chair of the AAC may nevertheless have influenced interpretation of the findings. The inclusion of an independent co-author, reflexive journaling, triangulation, and the use of participant quotations and quantitative results were intended to mitigate this possibility, but it cannot be fully eliminated. Furthermore, TO2015 represented a distinctive case characterized by an explicit commitment to athlete-centred governance. As a result, the findings may not be fully generalizable to MSEs operating under different governance structures or levels of athlete representation.

Limitations also exist in relation to sampling and data handling. When athletes' responses were “not applicable” they were treated as missing values and handled accordingly through pairwise deletion. While this may maximize data retention, sample size results varied across analyses. Additionally, the data were primarily collected in the Athletes' Village and participation was voluntary. Given accessibility constraints and athletes' self-selecting to participate, the results may not be representative of all athletes and may introduce the possibility of sampling bias.

Finally, the timing of data collection spanned both in-Games and post-Games responses. This could have resulted in variation in athlete perceptions, related to performance outcomes or retrospective evaluation. Despite these limitations, the study offers an extensive empirical snapshot of athlete experience within an MSE. The convergence of findings across the quantitative and qualitative analyses strengthens confidence in the overall interpretation of athlete experiences within the TO2015 environment.

## 9 Conclusion

This empirical audit of TO2015 examined whether an intentional, athlete-centred governance model was consistent with a high-quality experience for the prosumer population (i.e., athletes as prosumers). By assessing the formal integration of governance structures as they relate to perceived athlete satisfaction, this study repositions athlete-centredness as a meaningful attribute of an effective MSE. The findings of this study suggest that governance structures designed to increase athletes' influence in decision-making are feasible within an MSE context and may be associated with positive athlete experiences.

Furthermore, the exceptionally high degree of variance accounted for in the regression analyses suggests that service-related factors such as communication are closely associated with perceptions of athlete-centred satisfaction within the Games environment and should not be viewed solely as logistical components. In terms of the athlete as a prosumer, the performance-related conditions within the Games are intrinsically tied to the quality of services provided. These findings suggest a close relationship between athlete voice, athlete satisfaction, and the broader event environment. The descriptive patterns between rookie and veteran athletes highlight the limitation of a one-size-fits-all approach to governance in modern MSEs. While rookies appreciate novelty and discovery, veteran athletes view the experience from a performance perspective, expecting elite-level conditions as a baseline requirement. Consequently, pursuing an athlete-centred MSE may benefit from segmented delivery strategies that reflect these differing expectations.

Ultimately, this research offers a guide for future organizing committees to move beyond treating athletes as passive objects of service delivery. From a practical perspective, this includes coaches, sport organizations, and policymakers adopting governance structures that formally integrate athlete voice into decision-making processes. By adopting a stakeholder identification and salience framework that institutionalizes athlete power, MSEs may be better positioned to translate athlete-centred commitments into meaningful organizational reality rather than a rhetorical gesture. Despite evidence of a generally positive athlete experience within an athlete-centred governance environment, variation in service consistency highlights the importance of continuous monitoring and implementation fidelity. It should be noted that the cross-sectional design and reliance on self-reported data limit causal interpretation. Therefore, future research should explore how different models of athlete governance integration are associated with athlete outcomes, as well as the relationship between athlete experience and performance outcomes across MSE contexts.

## Data Availability

The raw data supporting the conclusions of this article will be made available by the authors, without undue reservation.
